# The Improvement of The Endogenous Antioxidant Property of Stone Fish (*Actinopyga lecanora*) Tissue Using Enzymatic Proteolysis

**DOI:** 10.1155/2013/849529

**Published:** 2013-02-03

**Authors:** Sara Bordbar, Afshin Ebrahimpour, Azizah Abdul Hamid, Mohd Yazid Abdul Manap, Farooq Anwar, Nazamid Saari

**Affiliations:** ^1^Department of Food Science, Faculty of Food Science and Technology, Universiti Putra Malaysia, 43400 Serdang, Selangor, Malaysia; ^2^Department of Food Technology, Faculty of Food Science and Technology, Universiti Putra Malaysia, 43400 Serdang, Selangor, Malaysia; ^3^Department of Chemistry, University of Sargodha, Sargodha 40100, Pakistan

## Abstract

The stone fish (*Actinopyga lecanora*) ethanolic and methanolic tissue extracts were investigated for total phenolic contents (TPCs) as well as antioxidant activity using 2,2-diphenyl-1-picrylhydrazyl (DPPH^•^) radical scavenging activity and ferric reducing antioxidant power (FRAP) assays. Both extracts showed low amount of phenolics (20.33 to 17.03 mg of gallic acid equivalents/100 g dried sample) and moderate antioxidant activity (39% to 34%  DPPH^•^ radical scavenging activity and 23.95 to 22.30 mmol/100 mL FeSO_4_ FRAP value). Enzymatic proteolysis was carried out in order to improve the antioxidant activity using six commercially available proteases under their optimum conditions. The results revealed that the highest increase in antioxidant activity up to 85% was obtained for papain-generated proteolysate, followed by alcalase (77%), trypsin (75%), pepsin (68%), bromelain (68%), and flavourzyme (50%) as measured by DPPH^•^ radical scavenging activity, whilst for the FRAP value, the highest increase in the antioxidant activity up to 39.2 mmol/100 mL FeSO_4_ was obtained for alcalase-generated proteolysate, followed by papain (29.5 mmol/100 mL FeSO_4_), trypsin (23.2 mmol/100 mL FeSO_4_), flavourzyme (24.7 mmol/100 mL FeSO_4_), bromelain (22.9 mmol/100 mL FeSO_4_), and pepsin (20.8 mmol/100 mL FeSO_4_). It is obvious that proteolysis of stone fish tissue by proteolytic enzymes can considerably enhance its antioxidant activity.

## 1. Introduction

Oxidative stress is recognized as the major cause of chronic diseases progression, such as cancer, hypertension, cardiovascular disease, stroke, arteriosclerosis, diabetes, and neurodegenerative disorders. This leads to the generation of highly reactive molecules which are responsible for the development of such diseases. Studies have revealed that the ingestion of antioxidant supplements or food containing antioxidants may reduce oxidative damages [1–3]. Natural antioxidants such as polyphenols, phytosterols, carotenoids, vitamins C and E, tocopherols, herbal extracts like rosemary, sage, and tea extracts have been commercialized as alternatives to synthetic antioxidants. In addition, proteins and protein hydrolysates derived from milk, soy, egg, and fish have also been shown to exhibit antioxidant activity [4, 5].

There is a great potential for the development of antioxidants from marine resources. Sea cucumbers are from those marine organisms that are considered as one of the most valuable sources of structurally diverse bioactive compounds with immense nutraceutical, pharmaceutical, and cosmeceutical potentials [6]. Sea cucumbers are crucial as human food source, particularly in some parts of Asia [7]. Several unique biological and pharmacological activities, namely, antiangiogenic, anticancer, antimicrobial, antitumor, and wound healing, are due to appreciable amounts of bioactive compounds found in sea cucumber species, including triterpene glycosides (saponins), chondroitin sulfates, sulfated polysaccharides, and peptides [6]. Some species of sea cucumbers were shown to contain potential antioxidant activity that may have been attributed by the presence of some bioactives such as flavonoids and bioactive peptides [8–13].

*Actinopyga lecanora *(Jaeger, 1833) from the phylum Echinodermata, class Holothuroidea, family Holothuriidae is a light gray or brown sea cucumber commonly seen on many of the South Asia sea shores. *A. lecanora* is commonly known as stone fish, because it looks like a smooth stone when disturbed, bloating up into a rounded, smooth shape and retracting its tube feet. This sea cucumber is classified among the edible species harvested for the food trade [14]. Well-founded data on medicinal properties and bioactive compounds of stone fish has not yet been reported. Thus, the current study is investigated to explore this multipurposes marine invertebrate as a potential source of antioxidants. The stone fish was quantitatively evaluated for total phenolic contents and its endogenous antioxidant activity. Besides, the improvement of antioxidant properties via generating proteolysates as well as bioactive peptides, using enzymatic proteolysis was appraised.

## 2. Materials and Methods

### 2.1. Enzymes

The enzymes used for enzymatic digestion, namely, alcalase and flavourzyme were supplied by Novozymes (Denmark), bromelain and papain were purchased from Acros Organic, pepsin from porcine gastric mucosa was supplied by Merck (Germany), and Trypsin from beef pancreas was supplied by Fisher Scientific (UK).

### 2.2. Chemicals

Chemicals used for the assays were of the analytical grade, including Folin-Ciocalteau purchased from Fisher Scientific (UK) and o-phthaldialdehyde (OPA) and 1,1-diphenyl-2-picrylhydrazyl (DPPH^•^) reagent purchased from Sigma-Aldrich (USA). Glutathione and 2,4,6,-tri(2-pycridyl)-s-triazine were purchased from Acros Organic.

### 2.3. Sample Preparation

Stone fish was purchased from Pantai Merdeka, in the Kedah state, Malaysia. The internal organs (stomach and intestine) were removed and rinsed with cold distilled water, then immediately frozen and stored at −80 °C until used.

### 2.4. Composition Analysis

The analysis of proximate compositions was carried out using the standard methods of AOAC (2002) [15]. Water content was determined using air oven method (100 °C for 5 h; AOAC number 950.46B) and ash using basic heating technique (550 °C for 5 h; AOAC number 920.15), and crude protein was determined by nitrogen combustion procedure (AOAC number 992.15) with conversion factor of *N* × 6.25. The amount of total lipids was determined gravimetrically after the Soxhlet extraction of dried samples with hexane. All measurements were performed in triplicate. 

### 2.5. GC/MS Analysis

The qualitative and quantitative analysis of the stone fish crude extract was carried out using a GCMS QP5050A Shimadzu system consisting of an autosampler equipped capillary GC interfaced with an EI ionization quadrupole mass analyzer with a mass range of 900 Da for identification purposes. Helium was used as carrier gas (1 mL/min), and the column used was a Zebron ZB-FFAP fused silica capillary column (30 m × 0.25 mm I.D. × 0.25 film thickness). The oven temperature was programmed as follows: the initial temperature was held for 5 min at 50 °C and then from 50 °C to 250 °C at a rate of 6 °C/min and maintained for 15 min. Injector temperature was set to 250 °C. The compounds were individually identified based on coelution and MS analysis, when possible peak relative retention times were adjusted with those of documented samples. Each determination was carried out in triplicate.

### 2.6. Total Phenols Determination

#### 2.6.1. Ethanolic and Methanolic Extraction

The phenolic extraction was carried out according to the method of Mamelona et al. [9], with some modifications. Briefly, sea cucumber was first freeze-dried and turned into powder. About 10 g of sample was mixed with 500 mL 80% ethanol, and the same amount was mixed with 80% methanol; both were stirred continuously at 4 °C for 24 h in the dark, to avoid likely oxidation of sensitive compounds due to light and high temperature. After 24 h, the supernatants were separated by centrifugation at 10,000 ×g for 5 min and filtered using Whatman filter paper. The solvents were removed using rotary evaporator. Extracts were immediately frozen and kept at −80 °C for further use.

Total phenolic contents of extracts were determined according to the method of Mamelona et al. [9], with some modifications, using the Folin-Ciocalteau phenol reagent. 100 *μ*L of extracts were transferred into the test tubes, and their volumes were made up to 500 *μ*L with deionized water. 1.25 mL of 20% aqueous sodium carbonate solution was added to the 250 *μ*L Folin-Ciocalteu reagent in a test tube and diluted 10 times with distilled water. The mixture was vortexed vigorously and held in the dark for 45 min at room temperature. The absorbance was recorded at 725 nm against a blank containing the same mixture except the sample that was replaced with deionized water. The total phenolic content was expressed as gallic acid equivalents (GAE) in milligrams per 100 gram of extract, using a calibration curve generated with 10–100 *μ*g of gallic acid standard solution (e.g., *μ*g/mL). All measurements were performed in triplicate.

### 2.7. Proteolysis of Sea Cucumber Protein

Freeze-dried sample (5 grams) was autoclaved at 121 °C for 15 min to inactivate endogenous enzymes and microorganisms. The autoclaved sample was then dialyzed for 28 h (12 h against distilled water and 16 h against reaction buffer) at 4 °C in the dark. After dialysis, sample was incubated in a water bath to reach the reaction temperature and mixed with proteolytic enzyme at a ratio of 1/100 (w/w, enzyme/substrate) and started to proteolyze under optimum condition of each enzyme (Table 1). First sampling was taken before adding enzyme as a negative control; the subsequent samplings were done at 1 h interval for the first 10 h of proteolysis and then once after 24 h. Enzyme was re-added at every 5 h during the proteolysis. The samples were then immediately boiled at 100 °C for 10 min in order to inactivate proteases. Proteolysates were then centrifuged at 10,000 ×g for 20 min to separate insoluble and soluble fractions. The supernatants were collected and stored in a freezer at −80 °C for further use.

### 2.8. Peptide Content Determination and Proteolysis Profiling Using o-Phthaldialdehyde (OPA)

The o-phthaldialdehyde (OPA) based spectroscopic assay was performed to measure proteolysis of sea cucumber tissue in bufferic solutions, as described by Church et al. [16] with some modifications. A fresh OPA solution was prepared as follows: 20 mg sodium dodecyl sulfate (SDS) was added to 762 mg sodium tetrahydroborate and the mixture adjusted to 15 mL with deionized water. 16 mg OPA dissolved in 400 *μ*L ethanol along with 40 *μ*L ß-mercaptoethanol diluted in 5 mL deionized water also added to the mentioned mixture and the whole mixture was adjusted to a final volume of 20 mL with deionized water. 40 *μ*L sample was added directly to 300 *μ*L of OPA reagent in a 96-well plate, mixed and incubated for 2 min at room temperature, and then the absorbance was measured at 340 nm using ELISA plate reader (Power Wave × 340, Biotek Inc.). The peptide content was measured based on glutathione calibration curve constructed in the range of 0–200 ppm. Adequate dilution was needed if the OPA value measured was over the linear range of standard curve. The test was carried out in triplicate. 

### 2.9. Antioxidant Activity

#### 2.9.1. 2,2-Diphenyl-1-picrylhydrazyl DPPH^•^ Radical Scavenging Assay

2,2-Diphenyl-1-picrylhydrazyl DPPH^•^ radical scavenging activity for both extracts and proteolysates was determined according to the method of Bersuder et al. [17]. A volume of 500 *μ*L of each diluted sample was mixed with 1 mL of 20 mM DPPH^•^ in 80% ethanol. The mixture was then kept at room temperature in the dark for 60 min, and the reduction of DPPH^•^radical was measured at 517 nm using UV-Vis Double Beam spectrophotometer (UVD 2950, Labomed, Inc.). The DPPH^•^ radical scavenging activity (%) was calculated as follows:

Radical scavenging activity (%):

(1) $$\begin{array}{c} \frac{\text{Absorbance}\;\text{of}\;\text{control}-\text{Absorbance}\;\text{of}\;\text{sample}\;}{\text{Absorbance}\;\text{of}\;\text{control}}\times 100. \end{array}$$

A lower absorbance of the reaction mixture indicated a higher DPPH^•^ radical scavenging activity. Gallic acid was used as a standard (positive control) for solvent extracts and glutathione for proteolysates. Distilled water was used as blank. The tests were carried out in triplicate.

#### 2.9.2. Ferric Reducing Antioxidant Power (FRAP) Assay

The procedure described by Guoa et al. [18] was followed. Briefly, the FRAP reagent contained 2.5 mL of a 10 mmol/L TPTZ (2,4,6-tripyridyl-s-triazine, Sigma) solution in 40 mmol/L HCl plus 2.5 mL of 20 mmol/L FeCl_3_ and 25 mL of 0.3 mol/L acetate buffer, pH 3.6. The reagent was prepared freshly and kept warm at 37 °C. Aliquots of 40 *μ*L sample supernatant were mixed with 0.2 mL distilled water and 1.8 mL FRAP reagent, and the absorbance of reaction mixture at 593 nm was measured spectrophotometrically after incubation at 37 °C for 10 min. The FeSO_4_ was used as the standard solution. The final result was expressed as the concentration of antioxidants having a ferric reducing ability equivalent to that of 1 mmol/L FeSO_4_. Adequate dilution was needed if the FRAP value measured was over the linear range of standard curve. The test was carried out in triplicate.

## 3. Statistical Analysis

Statistical analysis and the comparison between the groups were performed using one-way ANOVA followed by Tukey's test to identify differences between treatments at 5% significant level, using Minitab version 14 (Minitab Inc., State College, PA, USA). 

## 4. Results and Discussion

### 4.1. Proximate Composition

The proximate composition of freeze-dried stone fish is given in Table 2. The contents of moisture, ash, fat, protein, and fiber were 6.65, 15.32, 3.65, 69.71, and 4.97%, respectively. The protein content on dry weight basis was relatively higher than that obtained from different species in previous studies [19–21] (Table 2). Accordingly, stone fish can be considered as a good source of protein.

### 4.2. Identification of Antioxidative Compounds Using GC/MS

**Figure 1:**
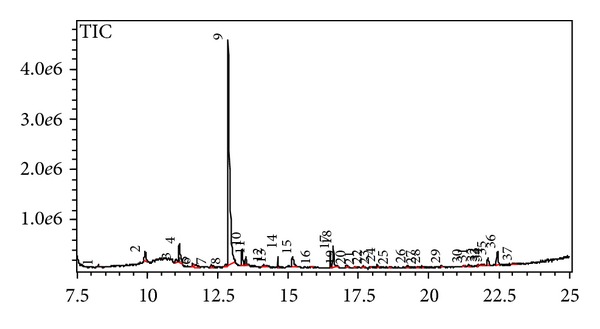
GC chromatogram of stone fish tissue extract. The detection limit for compounds identification is 10^−9^ g.

The composition of the antioxidative compounds present in crude extract of stone fish tissue was determined by GC/MS (Figure 1). Compounds were identified by comparing with the data base which were in agreement with Mass spectral library. Different array of compounds identified were hydrocarbons, sulfur-containing compounds, terpenes, acids, alcohols, and phenols, which contribute to the antioxidant activity of stone fish (Table 3). The major components present in the stone fish tissue crude extract included dimethyl sulfoxide (RT: 12.877, peak area: 59.7%), acetic acid (RT: 11.132, peak area: 4.2%) and dimethyl methylphosphonate (RT: 16.614, peak area: 4.1%). The main component of stone fish tissue extract with the highest peak area of 59.7% identified by GC/MS was dimethyl sulfoxide, which is an amphiphilic compound with ability to dissolve lipophilic compounds [22]. Dimethyl sulfoxide was reported as a potential source of antioxidant in recent studies [22, 23]. 

### 4.3. Antioxidant Activity and Total Phenols of Solvent Extracts

The natural extracts antioxidant properties are generally ascribed to redox reactions with some biocompounds present in the extracts such as phenolics, salt, sugars, carotenoids, ascorbic acid, and peptides in addition to some pigments such as gadusol. These compounds can neutralize free radicals by acting as rapid donors of a hydrogen atom to radicals [8–10]. Their presence in stone fish tissue was expected, since these natural molecules are relatively easy to assimilate. Moreover, main sources of sea cucumbers food are phytoplankton and particles derived from degrading marine macroalgae which are phenolic rich materials [8–10]. Therefore, the initial objective was to determine total phenolic content of the tissue extracts which could possibly contribute to the antioxidant activity.

The levels of total phenols from Atlantic sea cucumber, *Cucumaria frondosa* and three species of Malaysian sea cucumbers (*Holothuria scabra, Holothuria leucospilota, *and* Stichopus chloronotus*) have been reported by Zhong et al., Mamelona et al., and Althunibat et al. [8–10]. Based on the previous reports, most of the phenolic compounds in sea cucumber are hydrophilic compounds [8–10]. As solubility of antioxidants plays an important role in their antioxidative capacity, in this study the polar solvents including 80% ethanol and 80% methanol were chosen for the extraction of phenolics from stone fish. On the other hand, there is no single method that is able to provide a comprehensive and accurate profile of antioxidant activity, since different methods employ different action mechanisms such as termination of free radical mediated chain reaction, hydrogen donation, chelation of catalytic ions, and elimination of peroxides. Therefore, using multiple assays based on different antioxidant mechanisms would be crucial in providing a more reliable assessment of the samples antioxidant capacity [4, 24]. Two methods were applied in the present research to measure antioxidant activity of stone fish extracts, namely, 2,2-diphenyl-1-picrylhydrazyl (DPPH^•^) radical scavenging assay and the ferric reducing antioxidant power (FRAP) assay.

**Figure 2:**
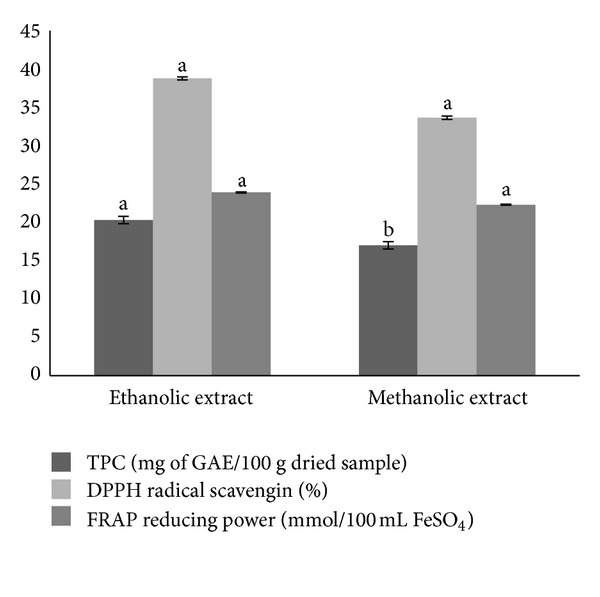
Total phenolic content (TPC) (mg of gallic acid equivalent (GAE)/100 g dried sample), DPPH^•^ radical scavenging activity (%), and ferric reducing antioxidant power (FRAP) assay (mmol/100 mL FeSO_4_) of ethanolic and methanolic extracts. Values were expressed as mean ± SD, *n* = 3. Mean value within each group with different letters (a, b) indicated significant difference at P ≤ 0.05.

Total phenols in the samples varied from 20.33 to 17.03 mg of gallic acid equivalents/100 g dried sample, depending on extracts involved (Figure 2), while the ethanolic extract showed significantly higher content of total phenolics (*P* ≤ 0.05). The DPPH^•^ radical scavenging activity was 39% for ethanolic extract and 34% for methanolic extract. The IC_50_ of extracts were found to be 1.97 mg/mL for ethanolic extract and 1.7 mg/mL for methanolic extract. There was no significant difference (*P* > 0.05) in the antioxidant activity of both extracts. However, both extracts showed significantly (*P* ≤ 0.05) higher inhibition concentration compared to gallic acid (0.05 mg/mL). The results from ferric reducing antioxidant power assay also showed no significant difference (*P* > 0.05) between the ethanolic and methanolic extracts (23.95 and 22.30 mmol/100 mL FeSO_4_, resp.). The results demonstrated a low contribution of phenolic compounds in antioxidant properties of stone fish extracts compared with other possible molecules such as carotenoids, ascorbic acid, proteins, and peptides such as glutathione. 

Based on the results, stone fish tissue with total phenols values ranging from 17.03 to 20.33 mg of GAE/100 g may not be considered as a rich source of phenolic compounds as well as endogenous antioxidants compared to those from plants which are known for their high phenolic content and antioxidant properties like fruits, vegetables, and medicinal plants with total phenolic content which varied from 169 to 10548 mg of GAE/100 g of dry sample [8–10]. However, the present findings are similar to those reported previously for other species of sea cucumber such as *Cucumaria frondosa*, *Holothuria scabra*, *Holothuria leucospilota*, and *Stichopus chloronotus*. [8–10].

With the aim of enhancing the antioxidant activity of stone fish tissue, the enzymatic proteolysis was carried out to digest the stone fish tissue proteins and generate proteolysates including bioactive peptides with higher antioxidative potentials.

### 4.4. Proteolysis Profiling and Peptide Content

Bioactive peptides play important roles in metabolic regulation and modulation and can be used as functional food ingredients, nutraceuticals, and pharmaceuticals to improve human health and prevent disease [25, 26]. Several studies have established that bioactive peptides have certain biofunctionalities that might serve therapeutic roles in the body systems [26, 27]. They may act as alternatives to small molecule drugs. Moreover, they provide lots of advantages over conventional small molecules due to their high biological activity and biospecificity to targets, wide array of medicinal properties, low levels of toxicity, structural diversity, and absence or low levels of accumulation in the body tissues [28–30].

Bioactive peptides are inactive within the sequences of the parent proteins and need to be released somehow to exert various physiological functions [28, 31]. The enzymatic hydrolysis method is the most commonly used in the food. The careful choice of a suitable enzyme and digestion conditions such as temperature and pH for the optimal activity of enzyme and the control of hydrolysis time are crucial for obtaining proteolysates with desirable functional and bioactive properties [4].

**Figure 3:**
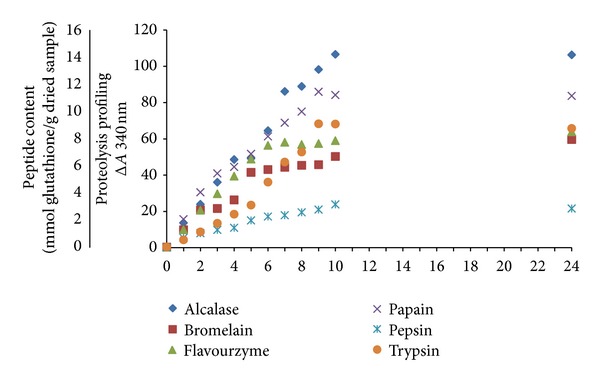
Proteolysis and peptide content (mmol/g dried sample) curves of stone fish proteolysates, generated by six enzymes under optimum conditions during 24 h of proteolysis. The OPA-peptide/protein complex absorbance was measured at 340 nm (mean ± SD, *n* = 3).

In the present study, the proteolysis of stone fish tissue with 6 proteases commonly used in enzymatic proteolysis of animal products, including alcalase, bromelain, flavourzyme, papain, pepsin, and trypsin was carried out under their optimum conditions for 24 h. The enzyme was re-added every 5 h, in order to complete the digestion of stone fish tissue proteins. The extent of proteolysis was measured using spectrophotometric assay by the o-phthaldialdehyde (OPA) method. The content of peptides generated during digestion was also measured, using glutathione as a standard for calibration curve. The proteolysis curves of stone fish proteins within 24 h of digestion along with peptide content are demonstrated in Figure 3. The proteolysis was characterized by higher rates for alcalase, papain, and flavourzyme. Bromelain also showed a rapid rate for the first 2 h, but subsequently the rate decreased. This could be due to the reduction in enzyme activity rather than completion of reaction, since it was again increased after re-adding enzyme after 5 h of proteolysis. On the other hand, proteolysis with trypsin started with a low rate while after re-adding enzyme the rate increased sharply. Again, it kept its constant increase by 9 h that it reached to the steady phase when the activity seemed to be finished due to the completion of reaction. Proteolysis with flavourzyme started with a high rate, and it constantly increased by 6 h, when it reached to the steady phase.

Under the operating conditions used in the present study, according to the maximum absorbance measured at 340 nm (Figure 3), alcalase was the most efficient protease to digest the proteins and produce peptides (14.18 mmol glutathione/g dried sample) while pepsin showed the lowest efficiency to digest the sample and produce peptides (3.15 mmol glutathione/g dried sample). The maximum amount of peptide content observed from each enzyme was in the following descending order: alcalase (14.18) < papain (11.43) < trypsin (9.06) < flavourzyme (8.49) < bromelain (7.92) < pepsin (3.15) mmol glutathione/g dried sample. The enzymatic reaction seemed to be completed after 10 h of digestion, since the curves reached the steady phase, when no apparent hydrolysis took place, except for bromelain, which slightly extended to 24 h.

The differences observed in the hydrolysis rate and pattern might be due to the differences in the properties of enzyme cutting sites as well as the accessibility of peptide bonds to each protease. Previous studies also revealed that the degree of hydrolysis of seafood materials using commercial enzymes was highly variable and depended on the nature of substrate, enzyme/substrate ratio and operating conditions [27]. 

### 4.5. Antioxidant Activity of Stone Fish Proteolysates

Antioxidant properties of proteolysates and bioactive peptides have been described in an increasing number of studies in recent years. A great deal of attention has also appeared to identify and assess antioxidative potential of bioactives derived from marine organism proteins and their possible applications as functional foods and nutraceuticals, to control various oxidative processes in the human body as well as in food [25–27, 31]. Since now, specific assays have not been developed or standardized to measure the antioxidative capacity of peptides or peptide mixtures. Therefore, assays that are commonly used for measuring antioxidative capacity of nonpeptidic antioxidants have been used in the literature to measure the antioxidative capacity of peptides as well [4]. In this study, we have reported our analytical results from DPPH^•^ radical scavenging activity and ferric reducing antioxidant power (FRAP) of stone fish proteolysates obtained from various enzymatic treatments. The objective was to improve the antioxidant property of stone fish tissue by the proteolysis of its proteins and production of proteolysates and antioxidative bioactive peptides. The changes in the antioxidant activity of proteolysates during the 24 h of hydrolysis were also observed.

#### 4.5.1. 2,2-Diphenyl-1-picrylhydrazyl (DPPH^•^) Radical-Scavenging Assay

One of the most common methods widely used to investigate the antioxidant capacity of natural compounds is 2,2-diphenyl-1-picrylhydrazyl (DPPH^•^) free radical scavenging assay which relies on the reduction of DPPH^•^ which is a stable free radical that shows maximum absorbance at 517 nm, in the presence of a hydrogen donating substances (antioxidants) [24].

**Figure 4:**
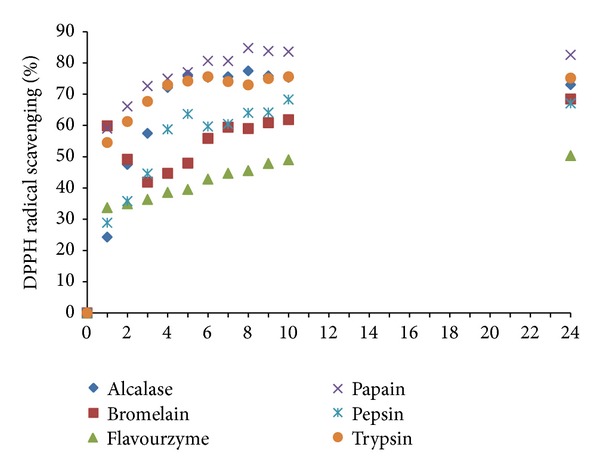
DPPH^•^ radical scavenging activity (%) of stone fish proteolysates derived from enzymatic digestion of stone fish tissue under optimum conditions for 24 h. Absorbance was measured at 517 nm (mean ± SD, *n* = 3).

The scavenging activity of stone fish protein hydrolysates for DPPH^•^ during 24 h of hydrolysis is shown in Figure 4. All treatments produced proteolysates with significantly (*P* ≤ 0.05) higher DPPH^•^ radical scavenging activities compared to the nonhydrolyzed sample. The radical scavenging curves of all proteolysates displayed a high rate for the first 1 h of hydrolysis. For alcalase and pepsin, the rate had a sharp increase by 5 h of hydrolysis, after which it decreased a bit and again increased until reaching to the steady phase. Proteolysates prepared by bromelain showed a high rate of activity for the first 2 h, but the activity dramatically decreased by 4 h. The decrease in radical scavenging activity might be due to the generation of oxidant peptides, since bromelain is an endoprotease which cut the generated antioxidative peptides to smaller size. Then, the second phase of activity was again increased before reaching the plateau. The percentage of DPPH^•^ scavenging activities of different proteolysates varied from 50% to 85%. Proteolysates prepared from papain showed significantly (*P* ≤ 0.05) the highest activity against DPPH^•^ (85%) followed by alcalase (77%), trypsin (75%), pepsin (68%), bromelain (68%), and flavourzyme (50%). It has been reported that the type of enzyme employed in protein hydrolysis process has an effect on the antioxidant activity potential due to the enzyme mechanism of action. Besides, several findings have also suggested that peptide size and solubility, the amino acid composition, sequence and abundance of free amino acids may have a key role in determining the DPPH^•^ radical scavenging capacity [1, 4, 31, 32]. Differences in assay conditions such as pH and/or the peptide sequences involved during the experiment have also been found to contribute to the differences in DPPH^•^ radical scavenging efficacies [31, 32].

**Figure 5:**
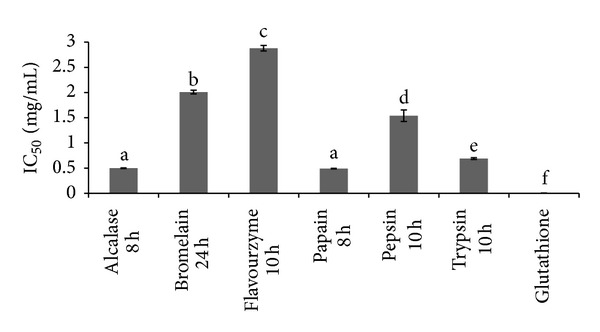
IC_50_ value of DPPH^•^ radical scavenging activity for stone fish proteolysates (mg/mL). Glutathione was used as positive control. a, b, c, d, e, and f indicated significant differences at the confidence level of *P* ≤ 0.05 (mean ± SD, *n* = 3).

IC_50_ (the concentrations of antioxidant substance scavenging 50% of DPPH^•^ radical) were determined for the proteolysates of each enzyme that showed the highest radical scavenging activity. The lowest IC_50_ value was obtained from both papain (0.49 mg/mL) and alcalase (0.5 mg/mL) proteolysates after 8 h of proteolysis, followed by trypsin (0.69 mg/mL after 6 h), pepsin (1.54 mg/mL after 10 h), bromelain (2.01 mg/mL after 24 h), and flavourzyme (2.88 mg/mL after 24 h). All proteolysates showed significantly (*P* ≤ 0.05) higher IC_50_ compared to glutathione (0.006 mg/mL) (Figure 5). 

Our results revealed that the proteolysis of stone fish with papain could considerably increase the DPPH^•^ free radical scavenging activity of tissue extract from 34–39% up to 85% on the other hand, IC_50_ decreased from 1.97–1.7 mg/mL of tissue extract to 0.49 mg/mL of papain proteolysate. Stone fish proteolysates obtained from alcalase and papain digestion were found to possess strong radical scavenging capacity compared to the similar marine sources such as marine rotrifer (46–50%) [33], sand eel (77%) [34], red tilapia (IC_50_ of 4.45 mg/mL) [35], albone viscera (IC_50_ of 4–7 mg/mL) [36] and smooth hound (IC_50_ of 0.6 mg/mL) [37].

#### 4.5.2. Ferric Reducing Antioxidant Power (FRAP) Assay

FRAP was used as the second assay for evaluating the ability of stone fish proteolysates to donate electron. FRAP assay uses antioxidants as reductants in a redox-linked colorimetric method, applying an easily reduced oxidant system present in stoichiometric excess. The principle involves the reduction of ferric tripyridyl triazine (Fe III TPTZ) complex to ferrous form (which has an intense blue color) at low pH which can be monitored by measuring the change in absorption at 593 nm [38].

**Figure 6:**
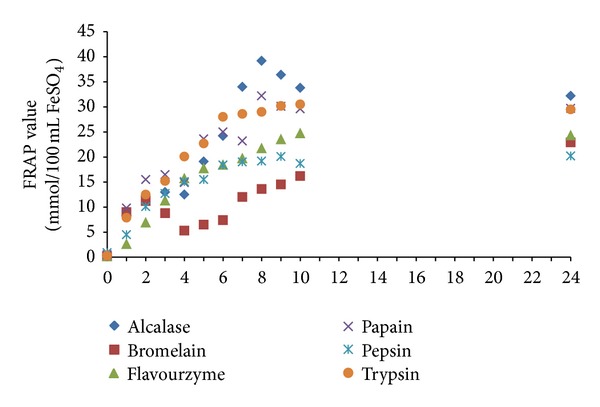
Ferric reducing antioxidant power (FRAP) of stone fish proteolysates derived from enzymatic digestion of stone fish tissue under optimum conditions for 24 h. Absorbance was measured at 595 nm, (mean ± SD, *n* = 3).

Several studies have reported a direct correlation between antioxidative activities and reducing power of protein hydrolysates from different bioresources [1, 18, 25, 36]. In this study, the ability of stone fish proteolysates to reduce Fe^3+^ to Fe^2+^ was determined. As displayed in Figure 6, the reducing power of all proteolysates increased as the proteolysis time increased up to 10 h. The highest activity was achieved in proteolysates produced by alcalase with FRAP value 39.2 mmol/100 mL FeSO_4_ obtained after 8 h of proteolysis followed by papain (29.5 mmol/100 mL FeSO_4_ after 8 h), trypsin (23.2 mmol/100 mL FeSO_4_ after 9 h), flavourzyme (24.7 mmol/100 mL FeSO_4_ after 10 h), bromelain (22.9 mmol/100 mL FeSO_4_ after 24 h), and pepsin (20.8 mmol/100 mL FeSO_4_ after 9 h), respectively. There were no significant changes (*P* > 0.05) when the hydrolysis time was further continued from 10 h to 24 h. Previous studies on ferric reducing antioxidant power (FRAP) of enzymatic proteolysates concluded that the reducing power activity was related to some factors including size and molecular weight of peptides, sequence of amino acids in peptides, number of hydrophobic amino acid, and amount of sulphur containing and acidic amino acids. Besides, the presence of some amino acids such as Leu, Lys, Met, Tyr, Ile, His, and Trp has been reported contributing to the strong reducing power of proteolysates [4, 25].

Comparing the reducing power activity of stone fish proteolysates with those medicinal plants which are known for their high antioxidant properties showed that stone fish proteolysates reducing power activity were similar to those medicinal plants with low FRAP value (1–5 mmol/L = 10–50 mmol/100 mL) [39]. However, they showed higher reducing power activity than fruits [18, 40], medicinal mushrooms [25], and other marine proteolysates [41] reported in previous studies.

Nevertheless, an increase in FRAP value of stone fish proteolysates was obtained from 22.30 to 23.95 mmol/100 mL FeSO_4_ of tissue extracts to more than 39.2 mmol/100 mL FeSO_4_ of alcalase proteolysates. The observed enhancement of both DPPH^•^ radical scavenging and FRAP value resulted directly from the generation of antioxidative bioactive peptides due to the enzymatic digestions and/or increased solvent exposure of some effective amino acids. While good antioxidant activity was observed from proteolysates, it is still not well understood how the composition of peptides influenced their ability to inhibit oxidation.

Comparing the results from DPPH^•^ radical scavenging activity and FRAP reducing power of proteolysates demonstrated some differences between these two assessment methods of antioxidant activity in some cases. Since the mechanism of antioxidative action measured and/or reaction conditions used are different from one assay to another, a test sample may show different results for antioxidative capacity depending on the assay system used. For example, a good ferric reducer may not show a high activity in radical scavenging assays. Moreover, the conditions of the assay may affect the antioxidative capacity. For example, the reducing capacity of an antioxidant can be affected by pH of the assay media. Solubility of antioxidants in the reaction media also plays an important role in their antioxidative capacity [1, 4]. In the present findings, moderate free radical scavenging and reducing power activities of crude alcoholic extracts of stone fish tissue were recorded, while those taken before adding enzyme including the bufferic tissue extracts showed no radical scavenging and very low reducing power activities. The reduction in antioxidant activity of bufferic extracts comparing to those alcoholic extracts could be due to the autoclaving process at high temperature along with dialysis, which led to the inactivation of some thermal sensitive and removal of water-soluble active constituents like enzymes, phenolics, flavonoids, sugars, salts, ascorbic acid, peptides, vitamins, and other endogenous antioxidative compounds from the sample.

### 4.6. Antioxidant Activity versus Proteolysis Time

The correlation between the degree of proteolysis for 24 h and the antioxidant activity of proteolysates measured by DPPH^•^ radical scavenging assay and FRAP value were determined. Results indicated strong correlations between both DPPH^•^ radical scavenging activity (*R* ^2^ > 0.8) and FRAP reducing power (*R* ^2^ ≤ 1) and degree of proteolysis. It could be suggested that enhancing the time of proteolysis led to the increase in the antioxidant activities. The increase in proteolysis time leads to the decrease in size of peptides. Therefore, proteolysates with smaller peptides may possess higher antioxidant activities. Some literature reports have shown a decrease in DPPH^•^ radical scavenging capacity of proteolysates with increasing proteolysis time [42, 43]. Some other studies have shown a marked increase in the DPPH^•^ radical scavenging activity with the extended proteolysis [44, 45]. Our results indicated that, after about 7 h of proteolysis, the rate reached the steady phase when the prolongation in enzyme activity has no significant effect on the radical scavenging activity. This is in line with the previous reports suggesting the increase of DPPH^•^ radical scavenging activity due to the extension of hydrolysis time [44, 45].

## 5. Conclusion

The bioactive peptides derived from marine resources with a wide array of biological activities and health beneficial effects have the potential to be exploited for nutraceuticals and functional food industry. The present study revealed that stone fish tissue could provide an appropriate antioxidative protection. These antioxidant activities increased even more after the digestion of tissue protein using enzymatic treatments, as a result of the production of bioactive peptides. Basically, the multifunctional nature of peptidic antioxidants, like having the ability to contribute other bioactivities, make them more attractive candidates than nonpeptidic antioxidants as dietary ingredients. The findings of the current research supported the Asian people beliefs about the health benefits of sea cucumbers in the folk medicine by approving the usefulness of stone fish biomolecules in preventing oxidation *in vitro*. This paper also highlighted the potential of stone fish-derived antioxidative proteolysates and bioactive peptides to be used in the food and beverages industries as natural alternative antioxidants to enhance the nutritional value and health benefits as well as the shelf life of the products.

## Figures and Tables

**Table 1:** Optimum conditions used for enzymatic proteolysis of stone fish.

Enzyme	Buffer (50 mM)	pH	Temperature (°C)
Alcalase	Borate buffer	7.9	55
Bromelain	Acetate buffer	4.5	50
Flavourzyme	Phosphate buffer	6.5	50
Papain	Phosphate buffer	6.5	55
Pepsin	Acetate buffer	3.6	37
Trypsin	Phosphate buffer	6.9	37

**Table 2:** Composition of freeze-dried stone fish tissue in comparison with other species.

Sea cucumber species	Water content (%)	Ash (%)	Fat (%)	Protein (%)	Fiber (%)	Reference
*Actinopyga lecanora *	6.65 ± 0.44*	15.32 ± 0.16*	3.65 ± 0.1*	69.71 ± 0.25*	4.97 ± 0.13*	Present study
*Holothuria scabra *	6–6.5	17.91–44.53	1.17–2.44	39.77–60.18	—	[19]
*Holothuria tubulosa *	8.28 ± 0.23	46.43 ± 0.51	0.71 ± 0.12	44.58 ± 1.01	—	[20]
*Holothuria polii *	10.23 ± 1.03	48.22 ± 1.02	0.55 ± 0.12	36.99 ± 0.62	—	[20]
*Acaudina molpadioides *	8.25	7.56	0.55	68.53	—	[21]
*Apostichopus japonicus *	21.55	21.09	1.85	55.51	—	[21]

All data were reported on dried basis.

*Mean ± SD, *n* = 3.

**Table 3:** Antioxidative compounds in stone fish crude extract detected by GC/MS.

Peak number	Compound name	RT*	SI* (%)	Peak area (%)
2	Diacetone alcohol	9.919	88	2.01
4	Acetic acid	11.132	100	4.2
9	Dimethyl sulfoxide	12.877	96	59.7
10	Boric acid	13.364	88	2.99
11	Propenoic acid	13.508	95	2.4
15	Butyl aldoxime	15.167	91	3.4
17	Propanedioic acid	16.509	73	1.69
18	Dimethyl methylphosphonate	16.614	76	4.1
22	phenol	17.692	81	0.28
35	Pyrrolidinedione	22.108	92	1.7
—	Other compounds	—	—	4.70

*RT: retention time.

*SI: similarity index.
